# Bilateral adrenal hemorrhage after hip arthroplasty: an initially misdiagnosed case

**DOI:** 10.1186/s12894-019-0536-7

**Published:** 2019-11-04

**Authors:** Lei Wang, Xiao-fei Wang, Ying-chao Qin, Jia Chen, Cun-hai Shang, Guo-feng Sun, Ning-chen Li

**Affiliations:** 1 Department of urology, Peking University Shougang Hospital, Peking University Health Science Center, Beijing, 100144 China; 2 Peking University Wu Jieping Urology Center, Peking University Health Science Center, Beijing, 100144 China; 3 Department of General Surgery, Shuangqiao Hospital in Chaoyang District, Beijing, 100024 China; 4 Department of Medical Imaging, Peking University Shougang Hospital, Peking University Health Science Center, Beijing, 100144 China; 5 Peking University Wu Jieping Urology Center, Peking University Shougang Hospital, 9# Jinyuanzhuang Road, Shijingshan District, Beijing, 100144 China

**Keywords:** Bilateral adrenal hemorrhage, Arthroplasty, Stress, Anticoagulants, Glucocorticoid replacement, Case report

## Abstract

**Background:**

Bilateral adrenal hemorrhage (BAH) is a rare but potentially catastrophic condition. Its clinical manifestation is often non-specific and sometimes difficult to be diagnosed in time.

**Case summary:**

A 57-year-old woman, who presented with severe fatigue, nausea and vomiting after left hip arthroplasty due to her femoral neck fracture in a local hospital, was transferred to our medical center. Laboratory results revealed significant hyponatremia, low serum cortisol and elevated serum ACTH. Computed tomography (CT) showed a bilateral adrenal mass, measured 3.6 × 2.7 cm on the left and 3.4 × 2.3 cm on the right. Further magnetic resonance imaging (MRI) confirmed the diagnosis of BAH. The patient was prescribed with oral prednisolone acetate, 5 mg, tid, and her condition improved gradually. Nine months after, the patient was in good condition with 5 mg prednisolone acetate per day. CT revealed a clearly shrunken adrenal mass compared with 9 months ago.

**Conclusions:**

This case illustrates the difficulty in making the diagnosis of BAH with atypical presentation. Such cases necessitate greater alertness on the part of the clinician and require rapid diagnosis and prompt glucocorticoid replacement for better clinical outcomes.

## Background

Adrenal hemorrhage (AH) is a rare condition with a reported incidence of only 5 in 1,000,000 [1]. Bilateral adrenal hemorrhage (BAH) is extremely rare and comprises only 10% of all AH cases. However, BAH is potentially fatal, carrying a mortality rate of 15% [2]. BAH is reported to be associated with many issues such as trauma, surgery, infection, use of anticoagulants, antiphospholipid syndrome (APS), and heparin-induced thrombocytopenia (HIT), etc. Abdominal pain, fever, nausea, vomiting and hypotension are common but nonspecific symptoms of BAH. Most of the symptoms are manifestations of adrenal insufficiency and acute adrenal crisis [3].

In this study, we describe a rare case of BAH after hip arthroplasty in a 57-year-old female with left femoral neck fracture. The stress of trauma and surgery, including the use of enoxaparin after surgery, should be its inducing factors. With nonspecific manifestation, this case was misdiagnosed for the first 2 weeks and then achieved a good recovery with treatment consisting of glucocorticoid replacement.

## Case presentation

A 57-year-old, previously healthy female suffered a serious car accident on May 3rd, 2018, which led to femoral neck fracture on her left side. Three days later, she underwent left hip arthroplasty in a local hospital. The patient was given enoxaparin (0.6 ml, ih. qd) for DVT prophylaxis on postoperative day (POD) one. The patient’s postoperative recovery was uneventful until POD 8, when she complained of severe nausea and vomiting, accompanied by vague epigastric pain, ceased defecation, decreased appetite, generalized weakness and fever with a Tmax of 39 °C. No acute hypotensive episode or hypoglycemia was recorded.

“Bowel obstruction” was first considered, and the patient was transferred to the general surgery department on POD 10 for parenteral nutrition and intestinal obstruction-related treatment. Her vital signs included temperature 38.7 °C, heart rate 104, respiration 19, blood pressure 120/74 mmHg. The abnormal physical examination included mild abdominal distention, slight tenderness of the upper abdomen, and slightly active bowel sounds. Laboratory examination revealed hypernatremia (152 mmol/L, normal 135–145 mmol/L) and hypopotassemia (3.2 mmol/L, normal 3.5–5.5 mmol/L).

**Fig. 1:**
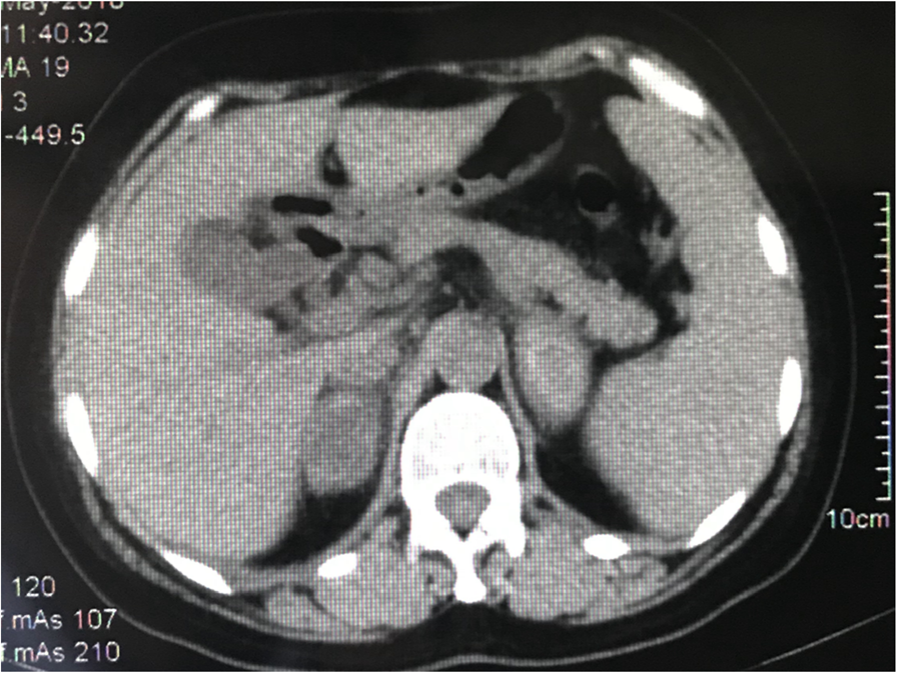
Axial computed tomography scan abdominal (postoperative day 12) revealed a bilateral adrenal mass with mixed high density

On POD 12, a computed tomography (CT) scan of the abdomen (Fig. 1) revealed a bilateral adrenal mass and a slightly enlarged spleen, but no signs of dilation or an air-fluid level within the intestine. However, the bilateral adrenal mass was thought to be adrenal adenoma and did not attract the properly deserved attention at that time. Consistent fluid resuscitation was given to correct low serum sodium and chlorine, and parenteral nutrition support was also given due to the patient’s bad appetite and vomiting.

**Fig. 2:**
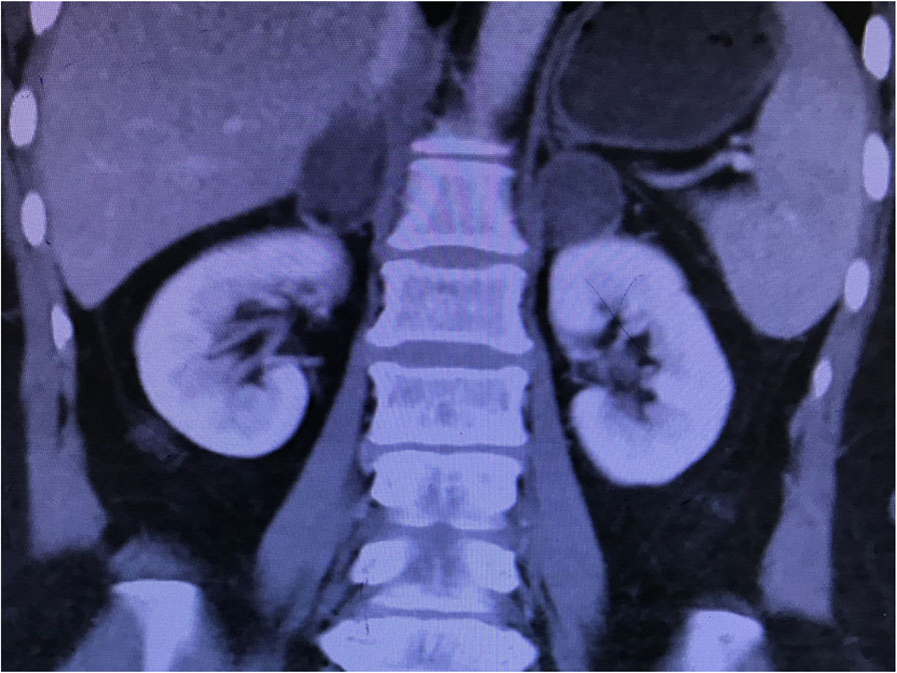
Enhanced computed tomography (coronal section) on postoperative day 26 showed a bilateral adrenal mass, measured 3.6 × 2.7 × 4.8 cm on the left and 3.4 × 2.3 × 4.2 cm on the right, with enhancement only seen in peripheral area of the mass

**Fig. 3:**
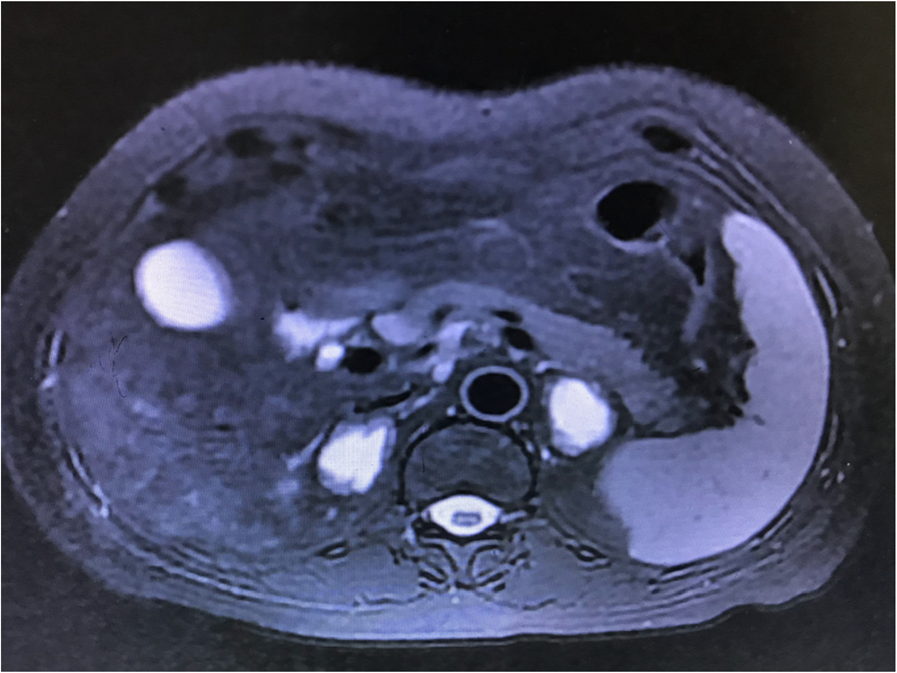
Magnetic resonance imaging (axial section, T2 weighed) on postoperative day 33 displayed water signal intensity of long T2 in central regions of bilateral adrenal mass

As the patient’s condition did not improve significantly, she was transferred to our hospital on POD 25, with very poor appetite, severe fatigue, nausea, vomiting, and general malaise. The physical examination was unremarkable, and her vital signs included temperature 36.1 °C, heart rate 62, respiration18, blood pressure 120/65 mmHg. The laboratory values revealed significant hyponatremia (120 mmol/L), hypochloremia (88 mmol/L, normal 99–110 mmol/L), compensatory metabolic acidosis (pH 7.36, BB − 3.4 mmol/L), elevated AST (75 IU/L, normal 13–35 IU/L), a low serum cortisol of 1.9 μg/dL (normal 3.7–19.4 μg/dL), and a high serum ACTH of 313 ng/L (normal 7.2–63.3 ng/L). Blood potassium, glucose, hematocrit, creatinine, platelet count and coagulation profile were all normal. CT scan revealed a bilateral adrenal mass, measuring 3.6 × 2.7 cm on the left and 3.4 × 2.3 cm on the right (Fig. 2). Differential diagnosis included primary adrenal cancer or metastatic tumor. As no historical result could be offered, whether the adrenal mass was new-onset or had existed for a long period was not clear. Magnetic resonance imaging (MRI) was advised and confirmed the most likely diagnosis to be bilateral adrenal hemorrhage (Fig. 3).

**Fig. 4:**
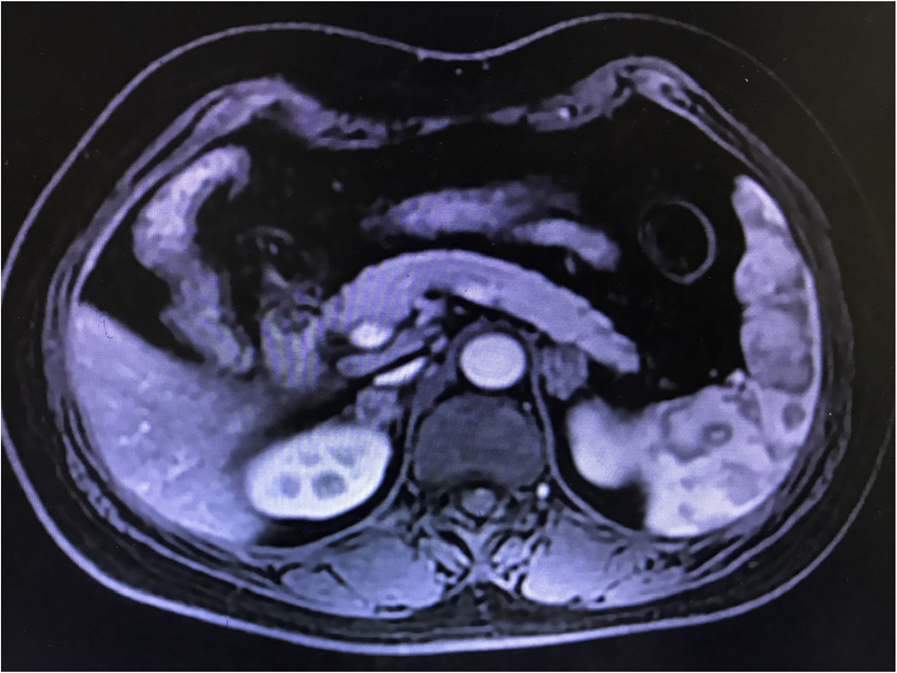
Enhanced magnetic resonance imaging (postoperative day 140) revealed a clearly shrunken adrenal mass compared with 3 months ago

**Fig. 5:**
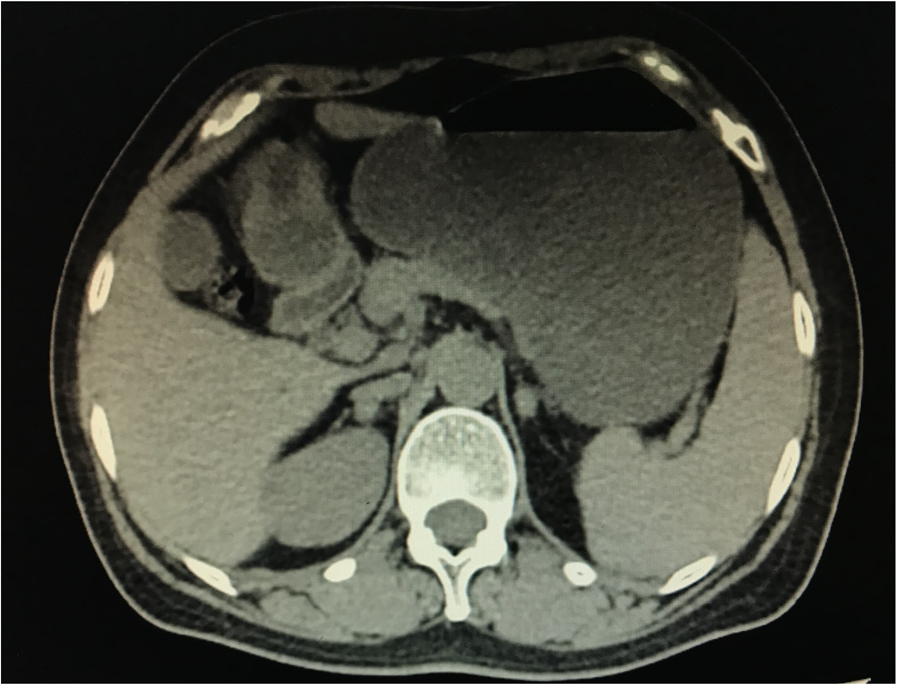
CT scan (9 months after discharge) revealed a continuously shrunken adrenal mass, measured 1.4 × 0.9 cm on the left and 1.3 × 0.9 cm on the right, compared with 9 months ago

Enoxaparin had been discontinued on admission, and the patient was started on oral prednisolone acetate, 5 mg, tid. Thereafter, the patient’s condition improved gradually, her nausea and vomiting disappeared, and her appetite and physical power also recovered significantly. The patient was in normal serum cortisol level at the time of discharge. The dose of oral prednisolone acetate was decreased to 5 mg, bid. She was also told that oral steroids might not be discontinued in the rest of her life. Three months later, the patient was in good condition, and the dose of oral prednisolone acetate was decreased to 5 mg, qd. In the patient’s latest follow-up 9 months after discharge, she still needed oral steroids replacement because of symptoms of adrenal insufficiency after complete withdrawal of the drug. MRI (Fig. 4) and CT (Fig. 5) images during her followup revealed a continuously shrunken adrenal mass compared with that during her hospitalization .

## Discussion and conclusions

BAH is a rare but potentially catastrophic complication. The reported inducing factors include trauma, surgery, anticoagulants, infection, myocardial infarction, chronic heart failure, APS, and HIT [4–8]. Cases without any of above risk factors have also been reported in previous literature [2, 3]. In this study, we report a rare such case in a middle-age female after hip arthroplasty. The most likely cause in this case is thought to be the use of enoxaparin, a common low-molecular-weight heparin used after surgery. Sharp stress from trauma and surgery is also considered to be a potential risk factor in this case. With the condition’s nonspecific clinical manifestation, the patient did not receive a correct diagnosis and timely glucocorticoid replacement for 2 weeks after episode of adrenal crisis in a local hospital. However, the patient’s recovery was rapid after BAH was diagnosed and oral prednisolone acetate was prescribed.

BAH after surgery is extremely rare. Mandanas S [9] had reviewed 36 such cases in the year of 2013. Herein, we made a further literature search and 12 additional cases published after the year of 2013 were summarized in Table 1 [6, 8–16]. The mechanism of AH has not been fully elucidated. Among potential mechanisms, the distinct vascular anatomy of the adrenal gland has been repeatedly mentioned [2, 7, 8]. The adrenal gland has an arterial network, and the blood flow is very fast. However, it only has a single vein, which leads to an abrupt transition of blood flow and renders the gland vulnerable to hemorrhage events. A sharp increase of arterial blood flow or a sudden thrombosis of the adrenal vein can all lead to AH. For example, stress-induced catecholamine increases adrenal blood flow, promotes platelet aggregation, and induces adrenal vein spasm, which results in the blood vessels being filled with a large amount of blood flow, the vascular wall being damaged and ruptured, and eventually leading to a bleeding. Other implicated factors include aging-related reduced capillary resistance in the adrenal vascular bed [15] and adrenal vein thrombosis in hypercoagulable states, such as antiphospholipid syndrome [7].

**Table 1:** Summary of the literature on postoperative bilateral adrenal hemorrhage since 2013

No.	Year	Age	Gender	Procedure	DVT prophylaxis	POD	Symptoms	Lab. exams	Treatment	Outcome	Ref.
1	2013	52	F	Right hip arthroplasty	LMWH	9	Ap, hypotension, vomiting, weakness	Hyponatremia, hyperkalemia	Hydrocortisone fludrocortisone	Adrenal insufficiency, oral replacement	[9]
2	2014	67	M	Laparotomy, adhesiolysis	Not shown	4	Ap, confused, pyrexia	Hyponatraemia, hypocalcaemia hypermagnesaemia	Hydrocortisone	Long-term fludrocortisone	[10]
3	2014	48	F	Right partial nephrectomy	LMWH (for PE)	6	Syncope, hypotension, lethargy, fever	Thrombocytopenia, hyponatremia	Hydrocortisone	Adrenal insufficiency, oral replacement	[11]
4	2015	45	F	Chole-cystectomy	LMWH	9	Ap, fever, nausea, vomiting	Hyponatremia, thrombocytosis, antiphospholipid antibodies(+)	Fludrocortisone	well	[6]
5	2015	65	F	Total knee arthroplasty	LMWH enoxaparin	8	Vague, epigastric pain, lethargy, fever, nausea	Hyponatremia, hypokalemia	Hydrocortisone	well	[12]
6	2015	75	M	Total hip replacement	Warfarin	14	Watery diarrhoea, vomiting, hypotension	Hyponatremia, hyperkalemia	Hydrocortisone	Adrenal insufficiency, oral replacement	[13]
7	2015	48	F	Total knee arthroplasty	Aspirin LMWH	8	Ap, fever, hypotension	Thrombocytopenia, heparin platelet factor 4 antibody(+)	Steroids	Well	[14]
8	2016	93	F	Hemi-colectomy	Not shown	4	Ap, hypotension, syncope	hyponatremia, hypokalemia, heparin platelet factor 4 antibody(+)	Hydrocortisone	Stable, lifelong steroids	[15]
9	2017	76	F	Pancreaticoduodenectomy	Not shown	5	Ap, hypotension, nausea, vomiting	Hemoglobin drop, hyponatremia, hyperkalemia	Hydrocortisone	well	[16]
10	2018	65	F	Colectomy	LMWH	4	Fever, fatigue, lethargy, hypotension	Hyponatremia, hyperkalemia	Steroids	well	[8]
11	2018	72	M	Total colectomy	LMWH	5	Anorexia, lethargy, hypotension, fever	Hyponatremia; anticardiolipin antibodies(+)	Hydrocortisone	well	[8]
12	2019	57	F	Left hip arthroplasty	LMWH enoxaparin	8	Ap, fever, nausea, vomiting, weakness	Hyponatremia, hypochloremia, metabolic acidosis	Oral predni-solone acetate	Adrenal insufficiency, oral replacement	Our case

*DVT* Deep venous thrombosis, *POD* Postoperative day, *Lab.* Laboratory, *LMWH* Low-molecular-weight heparin, *Ap* Abdominal pain, *PE* Pulmonary embolism

Heparin-induced-thrombocytopenia is thought to be another important factor. Though rare, AH has been reported repeatedly after the use of anticoagulants, including heparin, warfarin, dalteparin, doumadin, dabigatran, or enoxaparin [12, 17, 18]. K J Park [12] reviewed 16 cases of hip and knee arthroplasty patients who suffered from BAH. Anticoagulation prophylaxis was given in all of the cases as a routine modality to prevent deep venous thrombosis. Among them, HIT was identified as the cause of BAH after confirmatory HIT antibody tests in 7 cases. For our case, sharp stress from the femoral neck fracture and the subsequent hip arthroplasty surgery was a possible factor related to her BAH. We also postulate that the use of enoxaparin, whose relationship with BAH has only been reported once by K J Park [12] in 2015, could be a potential risk factor.

The clinical manifestation of BAH is often nonspecific and occurs as a result of hypocortisolism and hemorrhage, including symptoms such as abdominal pain, fever, nausea, vomiting, fatigue, weakness, confusion and hypotension. Acute adrenal insufficiency secondary to an adrenal hemorrhage, especially BAH, is severe and sometimes life-threatening. Thus, it requires prompt diagnosis and management to prevent death from primary adrenocortical insufficiency. In our case, the patient’s clinical manifestation was nonspecific and confusing, which led to a misdiagnosis in the first 2 weeks in the local hospital.

Diagnosis of adrenal hemorrhage is challenge due to its low incidence, the vagueness of its signs and symptoms, and its nonspecific blood test abnormalities. As the patient is in condition of acute adrenal insufficiency, many abnormalities could be confirmed in biochemical tests, such as low cortisol, elevated ACTH, and sometimes hyponatremia and hyperkalemia [19]. Noncontrast abdominal CT is thought to be a standard diagnostic assessment but is sometimes difficult to interpret in the differential diagnosis, especially when the CT is performed after the acute hemorrhage phase. MRI of the adrenal glands has higher accuracy in differentiating adrenal hemorrhage and has advantages over conventional CT because it can easily distinguish adrenal hematoma from adjacent necrotic tissue and determine the onset time of hematoma.

Adrenal crisis due to hemorrhage is very dangerous. The mortality rate of BAH can reach 15% even after treatment. If the diagnosis or starting time of proper treatment is delayed, the mortality rate may be higher [16, 19]. Intravenous hydrocortisone and rapid fluids are recommended to be given as initial treatment. Hydrocortisone could be administered with a 100 mg bolus and then 200 mg per day by continuous intravenous infusion. As for resuscitating the patient with fluid, saline can be administered. Long-term patients may require lifelong steroid replacement. For our case, as the course of hypoadrenalism had lasted for 2 weeks and the patient had tolerated the condition to some extent, we only offered oral hormone supplementation, and the patient achieved a rapid recovery.

To the best of our knowledge, our BAH case is special with an initial misdiagnosis for up to 2 weeks. This case indicates that it’s full of challenge in diagnosing a BAH with atypical clinical presentations. Such cases necessitate greater alertness on the part of the clinician and require rapid diagnosis and prompt glucocorticoid replacement for better clinical outcomes. Despite its rarity, due to its potentially fatal consequences, bilateral adrenal hemorrhage should be considered as a differential for acute deterioration of a patient’s condition after surgery.

## Data Availability

The datasets used and/or analysed during the current study are available from the corresponding author on reasonable request.

## Abbreviations

ACTH: Adrenocorticotropic hormone
APS: Antiphospholipid syndrome
BAH: Bilateral adrenal hemorrhage
CT: Computed tomography
DVT: Deep venous thrombosis
HIT: Heparin-induced thrombocytopenia
MRI: Magnetic resonance imaging
POD: Postoperative day
